# Application of convolutional neural networks to breast biopsies to delineate tissue correlates of mammographic breast density

**DOI:** 10.1038/s41523-019-0134-6

**Published:** 2019-11-19

**Authors:** Maeve Mullooly, Babak Ehteshami Bejnordi, Ruth M. Pfeiffer, Shaoqi Fan, Maya Palakal, Manila Hada, Pamela M. Vacek, Donald L. Weaver, John A. Shepherd, Bo Fan, Amir Pasha Mahmoudzadeh, Jeff Wang, Serghei Malkov, Jason M. Johnson, Sally D. Herschorn, Brian L. Sprague, Stephen Hewitt, Louise A. Brinton, Nico Karssemeijer, Jeroen van der Laak, Andrew Beck, Mark E. Sherman, Gretchen L. Gierach

**Affiliations:** 1 Division of Population Health Sciences, Royal College of Surgeons in Ireland, Dublin, Ireland; 2 Division of Cancer Epidemiology and Genetics, National Cancer Institute, Bethesda, MD USA; 3 Department of Pathology, Radboud University Medical Center Nijmegen, Nijmegen, the Netherlands; 4 Beth Israel Deaconess Medical Center, Harvard Medical School, Boston, MA USA; 5 University of Vermont and University of Vermont Cancer Center, Burlington, VT USA; 6 University of California, San Francisco, San Francisco, CA USA; 7 University of Hawaii Cancer Center, Honolulu, HI USA; 8 Department of Radiation Medicine, Hokkaido University Graduate School of Medicine, Sapporo, Hokkaido, Japan; 9 The University of Texas MD Anderson Cancer Center, Houston, TX USA; 10 Center for Cancer Research, National Cancer Institute, Bethesda, MD USA; 11 Mayo Clinic, Jacksonville, FL USA

**Keywords:** Cancer prevention, Cancer epidemiology

## Abstract

Breast density, a breast cancer risk factor, is a radiologic feature that reflects fibroglandular tissue content relative to breast area or volume. Its histology is incompletely characterized. Here we use deep learning approaches to identify histologic correlates in radiologically-guided biopsies that may underlie breast density and distinguish cancer among women with elevated and low density. We evaluated hematoxylin and eosin (H&E)-stained digitized images from image-guided breast biopsies (*n* = 852 patients). Breast density was assessed as global and localized fibroglandular volume (%). A convolutional neural network characterized H&E composition. In total 37 features were extracted from the network output, describing tissue quantities and morphological structure. A random forest regression model was trained to identify correlates most predictive of fibroglandular volume (*n* = 588). Correlations between predicted and radiologically quantified fibroglandular volume were assessed in 264 independent patients. A second random forest classifier was trained to predict diagnosis (invasive vs. benign); performance was assessed using area under receiver-operating characteristics curves (AUC). Using extracted features, regression models predicted global (*r* = 0.94) and localized (*r* = 0.93) fibroglandular volume, with fat and non-fatty stromal content representing the strongest correlates, followed by epithelial organization rather than quantity. For predicting cancer among high and low fibroglandular volume, the classifier achieved AUCs of 0.92 and 0.84, respectively, with epithelial organizational features ranking most important. These results suggest non-fatty stroma, fat tissue quantities and epithelial region organization predict fibroglandular volume. The model holds promise for identifying histological correlates of cancer risk in patients with high and low density and warrants further evaluation.

## Introduction

Among women, invasive breast cancer is the most commonly diagnosed female cancer in most countries worldwide.^1^ Increased mammographic breast density, which describes the radiologically appearing white tissue on a mammogram, is one of the strongest breast cancer risk factors^2^. A recent meta-analysis found that percent density, which reflects the proportion of total breast area comprised of dense fibroglandular tissue, is a stronger predictor of risk than absolute dense area.^3^ It is estimated that 43% of US women 40–74 years of age have dense breasts,^4^ but mechanisms accounting for the relationship between elevated density and breast cancer risk remain ill-defined.

Studies highlight that pre-cancerous lesions^5^ and breast tumors^6^ are more likely to occur in mammographically dense regions within the breast, suggesting the relevance of localized as well as global density measures in cancer development. The few studies that have examined histological correlates of breast density have suggested that higher breast density is associated with greater epithelial cell content and non-fatty stroma.^7,8^ While most studies to date have utilized quantitative microscopy to characterize breast tissue from women undergoing procedures for suspect lesions, one study of non-cancerous autopsy breast tissues also showed positive relationships between epithelial and non-fatty stromal tissue, particularly stromal collagen area, and percent density.^9^

Advancements in automated digital pathology now allow increased opportunities for characterization and quantification of breast tissue organization that can complement traditional microscopic assessments. Moreover, increasingly, studies are utilizing automated digital tools for complex tissue pathology assessment of breast cancer outcomes.^10,11^ The recent incorporation of progressive artificial intelligence platforms into digital pathology work systems now allows the utilization and expansion of these approaches to larger scale molecular epidemiological studies. Specifically, deep learning methods such as convolutional neural networks,^12^ are increasingly being employed for histological image recognition with high accuracy and reproducibility.^13–15^ We previously developed a deep learning convolutional neural network model for the assessment of tissue characteristics in hematoxylin and eosin (H&E)-stained whole slide breast tissue images,^16,17^ which classified whole slide images as epithelial, stromal and fat tissue. In this current study, we hypothesized that application of this model to whole slide images of H&E-stained fixed tissue specimens collected from diagnostic image-guided breast biopsies might enable identification of specific histologic correlates that underpin breast density, including both global and localized (peri-lesional) measures. Secondly, as more than 25 million women in the US have dense breasts,^4^ and because only a small proportion of these women will develop breast cancer, we also aimed to identify tissue correlates of breast density that may be important for distinguishing malignant from benign biopsy diagnoses separately among women with high and low breast density, to help inform cancer risk stratification among women undergoing a biopsy following an abnormal mammogram.

## Results

### Patient characteristics

Overall, patient characteristics were largely similar between the training (*n* = 588) and testing (*n* = 264) sets (Table 1). The mean age was 50 years, and most women were of white race (91.3%), college educated (82.3%), of normal weight (50.4%) and premenopausal (58.1%). Most mammograms were categorized after work-up as suspicious abnormality (BI-RADS diagnostic category 4: 83.7%). The remainder were categorized as probably benign (BI-RADS diagnostic category 3: 5.9%) or highly suggestive of malignancy (BI-RADS diagnostic category 5: 10.5%). A little over half of the core needle biopsies were ultrasound-guided (54.6%), with the remainder being stereotactic-guided (45.3%). Median global fibroglandular volume was 34.4%, and median localized fibroglandular volume was 40.0%. No difference was observed for global and localized fibroglandular volume between the training and testing sets. Among the *n* = 1036 biopsy targets, most biopsy diagnoses were benign (78.2%). Benign breast disease diagnoses were categorized according to benign non-proliferative (including non-proliferative fibrocystic change and other benign and discrete entities), proliferative without atypia (including ductal hyperplasia and sclerosing adenosis) and proliferative with atypia (including atypical ductal and lobular hyperplasia). Further, 8.0% of all biopsies yielded in-situ lesions, and 13.8% were invasive carcinoma (Table 1).

**Table 1:** Selected characteristics of study participants from the BREAST-Stamp Project, who were referred for an image-guided breast biopsy, stratified by the training and testing sets (*n* = 852)

Characteristic	Overall (*N* = 852)		Training (*N* = 588)		Testing (*N* = 264)		*P*-value*
	*n*	%	*n*	%	*n*	%	
Age at ipsilateral mammogram (years)							0.85
<45	175	20.5	119	20.2	56	21.2	
45–49	217	25.5	152	25.9	65	24.6	
50–54	202	23.7	142	24.2	60	22.7	
55–59	145	17.0	95	16.2	50	18.9	
60+	113	13.3	80	13.6	33	12.5	
Mean (SD)	50.8 (6.9)		50.8 (6.9)		50.7 (6.8)		0.91**
Race							0.81
White, non Hispanic	778	91.3	536	91.2	242	91.7	
Other	74	8.7	52	8.8	22	8.3	
Education level							0.70
<High school	14	1.7	9	1.6	5	2.0	
High school graduation	132	16.0	95	16.7	37	14.6	
College/graduation school degree	678	82.3	466	81.8	212	83.5	
BMI (kg/m^2^)							0.22
<25	427	50.4	283	48.4	144	54.8	
25-<30	212	25.0	153	26.2	59	22.4	
30+	209	24.7	149	25.5	60	22.8	
Mean (SD)	26.7 (6.3)		26.8 (6.2)		26.6 (6.5)		0.43†
Age at menarche (years)							0.41
≤12	326	38.9	216	37.2	110	42.8	
13	322	38.4	233	40.1	89	34.6	
14	114	13.6	80	13.8	34	13.2	
15+	76	9.1	52	9.0	24	9.3	
Age at first birth (years)							0.54
Nulliparous	189	22.3	134	22.9	55	21.2	
<25	269	31.8	193	32.9	76	29.2	
25–30	202	23.9	135	23.0	67	25.8	
30+	186	22.0	124	21.2	62	23.9	
Menopausal status							0.89
Premenopausal	472	58.1	326	58.2	146	57.7	
Postmenopausal	341	41.9	234	41.8	107	42.3	
Menopausal hormone therapy use							0.88
Never	719	86.1	497	86.0	222	86.4	
Ever	116	13.9	81	14.0	35	13.6	
First degree family history of breast cancer							0.45
0	636	77.0	439	77.6	197	75.8	
1	167	20.2	114	20.1	53	20.4	
2+	23	2.8	13	2.3	10	3.9	
Breast biopsy prior to enrollment							0.54
No	580	68.9	404	69.5	176	67.4	
Yes	262	31.1	177	30.5	85	32.6	
Global FGV (%)^c^							0.55
≤34.4 (%)	439	51.5	307	52.2	132	50.0	
>34.4 (%)	413	48.5	281	47.8	132	50.0	
Median (Range)	34.4 (0.6, 99.5)		34.4 (0.6, 99.5)		36.2, (1.4, 99.3)		
Localized FGV (%)^c^							0.21
≤40 (%)	406	50.7	289	52.2	117	47.4	
>40 (%)	395	49.3	265	47.8	130	52.6	
Median (Range)	40.0 (0, 100)		39.8 (0, 100)		43.3 (0, 100)		
Biopsy type							0.82^b^
Ultrasound-guided (14-guage)	445	52.2	309	52.6	136	51.5	
Stereotactic-guided (9-guage)	406	47.7	279	47.5	127	48.1	
Both	1	0.1	0	0.0	1	0.4	
BI-RADS mammography assessment							0.89
Probably benign finding	47	5.9	32	5.8	15	6.1	
Suspicious abnormality	670	83.7	463	83.4	207	84.2	
Highly suggestive of malignancy	84	10.5	60	10.8	24	9.8	
Pathologic diagnosis^a#^							0.23
Benign non-proliferative	282	33.1	190	32.3	92	34.9	
Proliferative without atypia	316	37.1	215	36.6	101	38.3	
Proliferative with atypia	57	6.7	44	7.5	13	4.9	
In-situ (LCIS or DCIS)	76	8.9	48	8.2	28	10.6	
Invasive breast cancer	121	14.2	91	15.5	30	11.4	
Characteristic (per biopsy target, *n* = 1036 biopsies)							
Biopsy type							0.63^b^
Ultrasound-guided (14-guage)	566	54.6	372	54.2	194	55.6	
Stereotactic-guided (9-guage)	469	45.3	315	45.9	154	44.1	
Both	1	0.1	0	0.0	1	0.3	
Pathologic diagnosis^#^							0.39
Benign non-proliferative	373	36.0	242	35.2	131	37.5	
Proliferative without atypia	369	35.6	242	35.2	127	36.4	
Proliferative with atypia	68	6.6	52	7.6	16	4.6	
In-situ (LCIS or DCIS)	83	8.0	53	7.7	30	8.6	
Invasive breast cancer	143	13.8	98	14.3	45	12.9	

*BMI* body mass index, *DCIS* ductal carcinoma in situ, *FGV* fibroglandular volume, *LCIS* lobular carcinoma in situ, *SD* standard deviation

Missing data were excluded from percentage calculations and statistical comparisons: 28 for education levels, 4 BMI, 14 age at menarche, 6 age at first birth, 39 menopausal status, 17 menopausal hormone therapy use, 26 first degree family history of breast cancer, 10 breast biopsy prior to enrollment, 51 percent volumetric local density (biopsy radius 0–2 mm), 51 BI-RADS mammography assessment

*P-values from Chi-Square test except where noted

**P-value from two-sample *t*-test

†*P*-values from Kruskal–Wallis test

^a^Among women with multiple biopsies, this was the worst pathologic diagnosis

^b^One woman from the test group who had both biopsy types was excluded from the Chi-square test

^c^The median cut points of breast density were determined among the training population and were consistent among all 852 women

^#^Benign non-proliferative diagnosis includes non-proliferative fibrocystic change and other benign and discrete entities; Proliferative without atypia includes ductal hyperplasia and sclerosing adensosis; Proliferative with atypia includes atypical ductal and lobular hyperplasia

### Associations between histologic features and breast density (global and localized fibroglandular volume)

As mentioned in the methods, 37 features were extracted from the output of the convolutional neural network model. Using these identified features in separate random forest regression models trained to predict global and localized fibroglandular volume, the correlations between predicted and actual fibroglandular volume measurements were 0.94 for global and 0.93 for localized fibroglandular volume, respectively. The top 10 correlates identified as most important for predicting both fibroglandular volume measurements are shown in Table 2, and the corresponding Gini index plots for global and localized fibroglandular volume are shown in Supplementary Fig. 2. Overall, similar features were identified as correlates of global and localized fibroglandular volume measures; however, some differences were noted. Normalized non-fatty stromal tissue quantity (i.e., stromal tissue quantity normalized to total breast tissue area on the whole slide image) and normalized fat quantity (i.e., fat tissue quantity normalized to breast tissue area on the whole slide image) were the strongest predictors of both global and localized fibroglandular volume. Of note, epithelium quantity did not rank among the top 10 features for global fibroglandular volume and was ranked 8th for localized fibroglandular volume. Features characterizing the spatial arrangement of the epithelial regions assessed using an area-Voronoi diagram^18,19^ were among the top 10 features ranked for prediction of both global and localized fibroglandular volume.

**Table 2:** Summary of top 10 ranked histologic features identified in the random forest model for the prediction of global and localized % fibroglandular volume (FGV)

Feature Name	Rank of feature importance Predicted model: global FGV (%)	Rank of feature importance Predicted model: localized FGV (%)
Global tissue amount		
Fat amount (µm^2^)	5	3
Fat amount normalized (%)	2	2
Stroma amount (µm^2^)	3	5
Stroma amount normalized (%)	1	1
Epithelium amount normalized (%)	−	8
Morphology		
Ecc epi regions (median)	7	−
Ecc epi regions (IQ)	6	9
Spatial arrangement of the epithelial regions (Area-Voronoi diagram)		
Voronoi area (mean µm^2^)	10	−
Voronoi area (median µm^2^)	−	−
Voronoi area (IQ µm^2^)	8	6
Ratio epi to Voronoi (mean)	−	7
Ratio epi to Voronoi (median)	−	4
Ratio epi to non-epi (mean)	9	−
Spatial arrangement of the epithelial regions (Delaunay Triangulation)		
Neighbors (SD)	4	10

*Ecc* eccentricity, *epi* epithelial, *IQ* interquartile, *FGV* fibroglandular volume, *SD* standard deviation

Only features ranked within the top 10 for prediction of each, FGV density measure, are included in the table

Features are ranked numerically and sequentially from 1 to 10, with 1 representing the most important feature and 10 representing the 10th most important feature

Sensitivity analyses were conducted to examine the influence of body mass index (BMI) and menopausal status on the predictions, and results from these investigations are detailed in Supplementary Table 2. BMI was consistently ranked as the strongest predictor of fibroglandular volume when included in the model. Interestingly, in this model, the normalized fat quantity was the next most important feature for both global and localized fibroglandular volume, followed by normalized non-fatty stroma quantity. When analyses were stratified by menopausal status, some differences in top ranking features were noted as outlined in Supplementary Table 2. For global fibroglandular volume prediction, the top-ranked features were similar; however, for localized fibroglandular volume, fat-related variables ranked lower among postmenopausal women than for premenopausal women.

### Exploratory investigation relating histologic features to biopsy diagnosis among patients with high and low fibroglandular volume

As elevated breast density is common among women,^4^ yet only a small proportion will develop invasive breast cancer, we aimed to identify histological correlates that could inform future breast cancer risk stratification among women undergoing diagnostic biopsy with either high or low breast density. The main objective of this exploratory investigation was to examine if the histologic features that were associated with cancer status were similar and/or different among women with low vs. high fibroglandular volume. Thus, using the 37 features, a random forest classifier was trained to predict invasive cancer vs. benign breast disease among women stratified into high or low fibroglandular volume (using the median cut-point of global (34.4%) and localized (40%) fibroglandular volume from the training population). The top-ranked features for predicting invasive cancer status separately among women with high vs. low fibroglandular volume are shown in Table 3. Firstly, features associated with the spatial arrangements of the epithelial regions were ranked most important (top two features) for predicting cancer status among women, irrespective of global fibroglandular volume (Table 3). H&E images highlighting examples of the top-ranked epithelial region spatial arrangement features, with corresponding mammograms from patients whose biopsies yielded diagnoses of atypical ductal hyperplasia and invasive carcinoma, are shown in Fig. 2a, b, respectively. Despite similar radiological global fibroglandular volume on both mammograms, the H&Es from each diagnostic biopsy, targeted to locally dense regions within the breast, reflect differences in the spatial arrangement of epithelium (Fig. 2a, b). Within Fig. 2, two features are highlighted: the mean and median area ratio of each epithelial region to its Voronoi region. Figure 2a represents a H&E whole slide image with low mean and median area ratio of each epithelial region to its Voronoi region. This slide has a diagnosis of atypical ductal hyperplasia and has both global and localized fibroglandular volume > median (global fibroglandular volume: 45%; localized fibroglandular volume: 61%). In contrast, Fig. 2b represents a H&E whole slide image with higher mean and median area ratio of each epithelial region to its Voronoi. This slide has a diagnosis of invasive carcinoma and has both global and localized fibroglandular volume > median (global fibroglandular volume: 49%; localized fibroglandular volume: 49%). Features of epithelial regions were also strongly associated with invasive cancer status in models stratified by localized fibroglandular volume. Among women with high localized fibroglandular volume, epithelial morphology features ranked as the most important (4 out of the top 5). Among women with low localized fibroglandular volume, epithelium quantity and the median number of epithelial regions were the top two ranked features, followed by normalized stroma quantity.

**Table 3:** Summary of top 10 ranked histologic features identified in the random forest model for the prediction of invasive cancer status among women with high and low % fibroglandular volume

Feature Name	High global FGV (%) (> median)	Low global FGV (%) (≤ median)	High localized FGV (%) (> median)	Low localized FGV (%) (≤ median)
Global tissue amount				
Fat amount (µm^2^)	−	8	−	5
Fat amount normalized (%)	−	3	−	7
Stroma amount (µm^2^)	−	9	−	−
Stroma amount normalized (%)	−	4	10	3
Epithelium amount (µm^2^)	4	−	4	1
Epithelium amount normalized (%)	6	−	7	8
Morphology				
Epithelial regions (IQ µm^2^)	−	−	1	4
Epithelial regions (max µm^2^)	9	−	−	−
Ecc epi regions (mean)	10	−	3	9
Ecc epi regions (median)	−	−	2	2
Ecc epi regions (IQ)	−	−	5	10
Spatial arrangement of the epithelial regions (Area-Voronoi diagram)				
Voronoi area (mean µm^2^)	5	7	6	−
Voronoi area (median µm^2^)	3	5	−	−
Voronoi area (SD µm^2^)	−	−	9	−
Voronoi area (IQ µm^2^)	7	6	−	−
Ratio epi to Voronoi (mean)	1	2	8	−
Ratio epi to Voronoi (median)	2	1	−	−
Ratio epi to Voronoi (IQ)	−	10	−	−
Ratio epi to non-epi (median)				6
Spatial arrangement of the epithelial regions (Delaunay Triangulation)				
Neighbors (mean number)	8	−	−	−

*Ecc* eccentricity, *Epi* epithelial, *IQ* interquartile, *FGV* fibroglandular volume, *SD* standard deviation

Only histologic features ranked within the top 10 for prediction of each density measure are included in the table

Features are ranked numerically and sequentially from 1–10, with 1 representing the most important feature and 10 representing the 10th most important feature

The median cut points of breast density used in stratification were: global FGV (%) 34.4, localized FGV (%) 40.0

The performance of the model for predicting invasive cancer among women with high vs. low global fibroglandular volume in the testing set is shown in Fig. 3a, b. An AUC of 0.92 (95% CI: 0.80–0.99) was achieved for predicting invasive cancer diagnosis among women with high global fibroglandular volume, and an AUC of 0.84 (95% CI: 0.71–0.94) was reached for predicting an invasive cancer diagnosis among women with low global fibroglandular volume. For cancer detection stratified according to high and low localized fibroglandular volume, similar prediction values were observed, as shown in Fig. 3c, d (high localized fibroglandular volume: AUC: 0.92 (95% CI: 0.79–0.99); low localized fibroglandular volume: AUC: 0.81 (95% CI: 0.65–0.96)). No significant differences were observed between the AUCs for high vs. low global (*p* = 0.24) or localized fibroglandular volume (*p* = 0.24).

## Discussion

We report that we can predict global and local mammographic fibroglandular volume by applying a deep convolutional neural network model to H&E-stained sections of image-guided breast biopsies prompted by an abnormal mammogram. Specifically, we show that greater non-fatty stromal and adipose tissue content and the spatial distribution of epithelial regions in tissues, rather than total epithelial quantities, were the strongest correlates of % fibroglandular volume. The cardinal histopathologic feature of breast cancer on low magnification is ‘invasion’, characterized by irregular epithelial growth with incursion of cells into normal structures. As anticipated, features extracted from the output of the convolutional neural network indicated that epithelial organization is the strongest correlate of invasive cancer irrespective of fibroglandular volume. Thus, we hypothesize that more complex analyses of dense tissue using convolutional neural networks or other imaging technologies may enable radiological recognition of textural patterns that reflect the epithelial disorganization characteristic of breast cancer. Recent preliminary analyses using convolutional neural networks suggest the potential of this approach.^20^

Our findings agree with prior literature using quantitative microscopy^9^ to understand histological correlates of breast density. Similarly, our findings support prior studies that suggest radiological density is largely non-fatty stroma, with relatively little variation in epithelial content by mammographic density.^8,9^ Further, we showed that other quantitative measures of fat tissue were also highly ranked as being important for the prediction of % fibroglandular volume. The heterogeneous nature of the top-ranked histologic features further supports the complexity of quantitative measures of breast density. A novel finding of our study was the identification of the spatial arrangement of epithelial regions as ranking among the top 10 correlates of fibroglandular volume. To define spatial arrangements, we used an area-Voronoi diagram and Delaunay triangulation, which are approaches that would be very difficult to reproduce using visual assessment. Voronoi decomposition is a method whereby an area is partitioned into smaller areas that surround regions that are closest to pre-specified points.^19,21^ In essence, our results suggest that tissues that display a high ratio of epithelial area to its corresponding areas of influence are characteristic of cancer in both high and low global fibroglandular volume contexts. The identified Voronoi area along with the area ratio of each epithelial region to its Voronoi region ranked among the top 10 correlates for both global and localized fibroglandular volume measures.

Beck and colleagues were among the first to highlight the potential of digital image analysis for examining histological features of breast cancer. They developed and utilized C-Path (Computational Pathologist), a machine learning tool, which identified features of stromal morphology that were especially important for predicting breast cancer prognosis.^10^ Although prognosis was not the focus of our analyses, using a similar approach, we also found that the quantity of non-fatty breast stromal tissue was among the top-ranked predictors of fibroglandular volume, supporting the contributory role of stroma to fibroglandular volume. This study highlights the importance of examining the tissue microenvironment of dense tissue in more detail, including conducting in-depth analysis of stromal components^17^ including collagen.^22,23^

A major clinical challenge is differentiating between the non-fatty stroma and at-risk epithelium that together constitute the ‘white’ dense areas that appear on a mammogram. Thus, despite similar measures of breast density for a radiologically dense breast, there could be considerable heterogeneity of tissue composition within the dense regions. As density alone may not be capable of defining epithelial organization, other techniques are needed. Potential solutions could be alternative imaging or further classification of density using neural networks.^20^ Findings from our exploratory analysis relating histologic correlates to biopsy diagnosis highlight the interindividual heterogeneity that may be apparent at the histological level despite having comparable radiological densities. Interestingly, we found that irrespective of fibroglandular volume, spatial arrangement of epithelium was the most predictive of a cancer diagnosis, showing that deciphering composition of the mammographic fibroglandular volume is important for identifying abnormalities at the histological level. Of note, the performance of the model was better in detecting cancer status among women with high fibroglandular volume (both global and localized) than among women with low fibroglandular volume, though this difference was not statistically significant. This could be an artifact of the model, i.e., a challenge of recognizing spatial patterns in low density. However, this finding could also support the concept of epithelial-stromal interaction in the progression of invasive cancer. Understanding the heterogeneity^24^ and significance of the epithelial region spatial arrangement and organization may provide important etiological clues for tumorigenesis, and additional assessment of these features is needed to examine their relationships with other epithelial histological risk markers including terminal duct lobular units.^25^

Since the publication by Beck and colleagues, there have been substantial advancements in digital pathology methodology, particularly with the advent of deep learning. For example, our investigation complements and expands on existing studies that have highlighted the potential of deep learning for identifying factors associated with breast cancer diagnosis.^14,15,26–28^ The publication of the CAMELYON16 challenge winners showed the ability of deep learning algorithms to detect lymph node metastasis with high accuracy with a comparable AUC to that obtained following pathological assessment (AUC = 0.96).^15^ While our limited sample size and the cross-sectional nature of the study design prevented detailed investigation of features associated with breast biopsy diagnoses, our preliminary findings also support the need for further investigations of biopsy tissue using deep learning algorithms.

This study has many important clinical implications and considerations. Firstly, the ability to make predictions using feature assessment alone and without the inclusion of additional breast cancer risk factor information suggests the utility of deep learning approaches for the clinical setting. However, to investigate potential influences of patient characteristics, we conducted sensitivity analyses. As expected given its well-established strong inverse association with % fibroglandular volume,^29^ BMI was the highest ranked feature for predicting % fibroglandular volume for models in which it was included. While recognition of clinical and participant characteristics is important, the inclusion of such factors in analytical models may mask lesser associations identified by the random forest approach. Second, clinically relevant histological features of biopsy tissue accompanied with radiological information may be of benefit to integrate into breast cancer risk models,^30^ which are increasingly being used in clinical practice for determining risk of invasive breast cancer. Our findings are of particular relevance for women with elevated breast density, who have had a prior breast biopsy, and as such are at elevated risk of developing invasive breast cancer. We aim that by identifying validated histological features at the time of clinical biopsy following an abnormal mammogram, we may be able to discriminate women at highest risk. Increased efforts are ongoing to include histological information, as well as mammographic density, in risk prediction tools as evidenced by the BCSC-BBD model.^31^ However, these current risk models do not yet incorporate detailed histology in risk estimates. The integration of biopsy histological features to current risk models that assess radiological and risk factor information may ultimately improve risk assessment and inform clinical management strategies by providing additional risk information on the increasing number of women undergoing breast biopsies after a mammogram. Furthermore, the application of deep learning models that can utilize histological breast biopsy features to predict future risk of breast cancer among women with dense breasts will be important among the growing population of women who experience an initial benign breast biopsy diagnosis. Future expanded studies will address these questions.

Our study has many strengths. Firstly, this analysis is one of the largest breast tissue studies to date to apply convolutional neural network models for the identification of tissue correlates of mammographic breast density. Further, from a biological mechanistic perspective, the ability to examine relationships between breast tissue features and localized fibroglandular volume measures allows the additional assessment of characteristics of the microenvironment of the suspect lesion, particularly factors that cannot be quantified by visual assessment but that may be important markers of cancer. Of note, we observed similarities in the top identified histologic correlates of both global and localized % fibroglandular volume, supporting the utility of biopsy tissues in understanding the global breast milieu. Further strengths of this study included the use of deep learning for delineating characteristics of tissue organization as well as for quantification of tissue components. Additionally, the utilization of diagnostic H&E whole slide images supports investigations of samples that are routinely collected during the clinical investigation following a biopsy, which suggests this approach may have clinical applicability and could compliment routine diagnostic assessment. This study related volumetric measures of breast density, determined from FFDM images, to 2D histological images from FFPE tissues, providing an important step toward a novel and complex approach to understanding breast cancer lesions and their relationships with breast density. Additional understanding of volumetric breast density would be gained by examining the 3D architecture of the BBD and breast cancer diagnoses. For example, future studies that incorporate volumetric density measures from 3D imaging modalities along with fresh tissues will provide a complementary extension to these findings.

However, this study also has limitations. While random forest approaches are effective in deciphering which histological features contribute most to model prediction, they do not yield easily quantifiable results for strengths of association. Our investigation of deep learning approaches to identify histologic features associated with cancer among women with high versus low breast density, while promising, was hampered by sample size. In our current sample set, the number of cancer cases within the testing dataset was limited in order to maximize the reliability of model training. Thus, additional, larger prospective studies are needed to identify biomarkers for cancer risk stratification among women with high breast density who may be referred to diagnostic biopsy following an abnormal mammogram. While the BREAST-Stamp participants are a representative sample of the population of women undergoing diagnostic investigation after an abnormal breast imaging exam, the women enrolled within the study were primarily white (91.3%), which is reflective of the catchment area of the University of Vermont Cancer Center. Further, detailed information on lifestyle factors including alcohol consumption and smoking were not available for the full study population in this analysis. Thus, additional studies among more diverse populations are warranted to determine the generalizability of study findings and to determine whether tissue correlates of mammographic density vary by race and also by lifestyle breast cancer risk factors. In addition, our analysis was restricted to H&E-stained tissue sections. While using H&E sections is important as they are clinically meaningful and routinely prepared following biopsy, investigation of features associated with complementary histological stains to characterize the breast microenvironment may also be informative. An additional consideration is the applicability of this approach to other populations. This investigation included breast tissue sections from a single cross-sectional study, for which standardized protocols were followed for specimen preparation, tissue sectioning and staining, and were completed in the same laboratory at the University of Vermont Medical Center. While this rigorous methodology reduced potential variability in the tissue samples being assessed, it may limit the generalizability of the findings. The approach applied in this current study used extensive contrast and color augmentation during training. This method increases the robustness of the deep learning model against staining variations, but may not be sufficient when dealing with external datasets with significant staining variations. Therefore, additional validation studies are needed that include tissue sections prepared in multiple laboratories. Such studies would be highly informative for determining the robustness of deep learning within diverse pathological clinical settings.

In conclusion, we highlight the potential of applying convolutional neural network models to digital pathology to gain insights into histological correlates that correspond to radiologic measures of breast fibroglandular volume, and to cancer risk. In doing so, in a population of women undergoing diagnostic breast biopsy, we found that epithelial organization was the strongest correlate of invasive cancer irrespective of fibroglandular volume. In addition, we found in agreement with prior studies that fat and non-fatty stromal features were important determinants of radiologic fibroglandular volume. As radiologic density alone may not be capable of defining epithelial organization, these findings suggest opportunities for future efforts using neural networks for enhanced capture of novel histologic as well as breast imaging features that may advance our understanding of breast tumorigenesis.

## Methods

### Study population

This study included women referred for diagnostic image-guided breast biopsy after an abnormal breast imaging exam between October 2007 and June 2010 at the University of Vermont Medical Center, and were enrolled as part of the National Cancer Institute’s (NCI) cross-sectional, molecular epidemiologic Breast Radiology Evaluation and Study of Tissues (BREAST)-Stamp Project. Details of the BREAST Stamp Project and study eligibility characteristics have been described previously.^25,29,32^ Eligible participants were women aged 40–65 years referred for image-guided biopsy who did not have breast implants, had not been diagnosed with breast cancer or received cancer treatments, had not undergone breast surgery within one year and had not received chemoprevention. During the enrollment period, mammography registry data indicated that 1227 patients met these eligibility criteria. Information supplied by the radiology facility included final assessment of the mammogram, in BI-RADS categories: 3, “probably benign finding”; 4, “suspicious abnormality”; and 5, “highly suggestive of malignancy”.^33^ A standard health history questionnaire which assessed established breast cancer risk factors was collected at the time of the mammogram,^34^ and upon providing consent to be enrolled in the study, additional detailed breast cancer risk factor information was collected by the research coordinator.^29^ The distribution of the collected breast cancer risk factor information, including the demographic and lifestyle characteristics of the enrolled BREAST Stamp study population, has been previously described.^25,29,32^ Details of the analytical population included in this current analysis are outlined in more detail below and described in Table 1. The Institutional Review Boards at the NCI and the University of Vermont approved the protocol for this project for either active consenting or a waiver of consent to enroll participants, link data and perform analytical studies.

### Breast biopsy specimens

Breast tissues obtained from ultrasound-guided core needle (14-gauge) or stereotactic-guided vacuum-assisted (9-gauge) biopsy, were routinely processed, and representative H&E-stained breast tissue sections were obtained from the formalin-fixed paraffin-embedded target blocks for each biopsy and, when collected during biopsy, from non-target blocks representing surrounding non-target tissue. The diagnosis was confirmed following pathological report review. For women who had ≥ two unilateral biopsy targets, the two targets with the most severe diagnoses were selected. If there were ≥ two bilateral targets, then one target from each breast was selected, sampling the tissues with the most severe diagnoses. H&E-stained breast biopsy tissue sections were digitized at ×20 magnification using the Aperio (47.7%) or Hamamatsu scanning systems (52.3%).

### Assessment of breast density

Assessment of breast density was conducted at the University of California, San Francisco on pre-biopsy raw digital mammograms from full-field digital mammography systems.^25,29,32,35,36^ Briefly, quantitative global^29^ and localized^25^ fibroglandular tissue volume (cm^3^) measures were determined using craniocaudal mammograms of the ipsilateral breast, taken at the time-point prior but nearest to the biopsy date. Percent (%) global fibroglandular volume was estimated using Single X-ray Absorptiometry, which utilized a breast density phantom attached to the compression paddle of the mammography machine.^25,29,32,35,36^ For the assessment of % localized peri-lesional fibroglandular volume measurements, the biopsy location and radius were identified on the pre-biopsy mammogram by the study radiologist.^25^ Localized % fibroglandular volume measurements at a volume ~0–2 mm^3^ surrounding but excluding the biopsy target location were utilized in this analysis.

### Analytical population

**Fig. 1:**
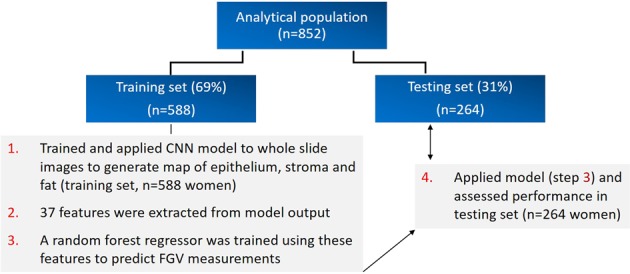
Workflow overview utilizing training and testing sets for the prediction of global and localized fibroglandular volume (FGV) measures from identified convolutional neural network model features

Of the women eligible for this study, 882 (69%) had Single X-ray Absorptiometry fibroglandular volume results available for the ipsilateral breast within the year before their breast biopsy. Of these, 852 women had target and non-target H&E slides from 1036 breast biopsies available for assessment. For convolutional neural network model training and assessment, as outlined in more detail below, the study population was randomly subdivided into a training dataset (*n* = 588; 69%) and a testing dataset (*n* = 264; 31%). Overall, the 588 women in the training set had 687 biopsies which encompassed 1587 H&E stained sections (667 from the target and 920 from the non-target blocks). For the testing group of 264 women, there were 349 biopsies (454 sections from non-target blocks). An overview of the study design is shown in Fig. 1.

### Development of the deep learning convolutional neural network model

Using the digitized H&E whole slide images from 588 women included in the training set, a deep convolutional neural network was trained to generate maps of tissue composition that classified whole slide images as epithelial, stromal and fat tissue.^16,17^ For model training, both target and non-target slides were included. The trained model was an 11-layer fully convolutional VGG-like network, a neural network architecture developed by Oxford’s Visual Geometry Group (VGG).^37^ The performance of the convolutional neural network model for generating whole slide image maps of epithelial, stromal and fat tissue has been outlined previously,^16^ and an example of the classification is shown in Supplementary Fig. 1. Briefly, the initial classification of the breast tissue (epithelial, stromal and fat composition) was completed through training of the convolutional neural network model based on manual annotation of these regions in 100 whole slide images, by trained students; these annotations were furthered reviewed by a pathologist. The AUC of the model for the classification of the breast tissue was 0.95.^16^ Following the generation of the whole slide image maps, features were extracted from the output of the convolutional neural network. These features were grouped into three main categories, describing global tissue quantities, the morphology of the epithelial regions, and spatial arrangements of epithelial regions. To examine spatial arrangements of epithelial regions, region adjacency graphs were used including area-Voronoi diagrams and Delaunay triangulation.^18,19^ The area-Voronoi diagram was utilized in the context of spatial distribution analysis to define areas of influence of epithelial regions in the image. Given a set of segmented epithelial regions *A*_*1*_*,…,A*_*n*_ in a whole slide image, the area-Voronoi of a region *V*_*a*_ *(A*_*i*_) is defined as the set of pixels in the image from which the distance to *A*_*i*_ is less than or equal to any other regions in the image. Overall, 37 features were extracted within these three categories; a description of the 37 features and their distributions in the training and testing sets are shown in Supplementary Table 1.

### Statistical analysis

Patient characteristics were compared between the training and testing sets using chi-square or Fisher’s exact tests for categorical variables and Wilcoxon rank sum tests for continuous variables. Using the 37 features extracted from the output of the convolutional neural network, a random forest regression model was used to predict global fibroglandular volume (%) and a separate random forest model was used to predict localized fibroglandular volume (%) (i.e., in the region of the biopsy target). The scikit-learn^21^ Python method was used for training of the random forest models. These models were then applied to the independent testing set to predict the fibroglandular volume measures. We chose random forests as this approach can account for any non-linear relationships between the features and has been shown to work well even when the number of features exceeds the number of observations.^38^ The output from the random forest model includes the Gini index plot as a measure of the predictive importance of the features. Supplementary Fig. 2 shows the Gini index results for features associated with global and localized % fibroglandular volume. Relationships between the predicted and radiologically quantified (actual) fibroglandular volume measures were assessed using Spearman rank correlations (r). Several sensitivity analyses examined the potential influence of participant characteristics known to be associated with fibroglandular volume on observed findings: (a) we additionally included body mass index (BMI) in the random forest regression model; and (b) we stratified analyses by menopausal status. We also assessed the potential influence of histologic features that were strongly correlated with each other in the prediction model. For highly correlated feature pairs (Spearman correlation: *r* ≥ 0.85), one feature was randomly selected to be excluded from the model. We then retrained the random forest models on the remaining 25 features. We also used the 25 features to separately predict each fibroglandular volume measure. When the number of features in the prediction model was reduced to include only one from among highly correlated features, the top selected features for fibroglandular volume prediction were similar; therefore, we present results from random forest analyses including all 37 features.

In an exploratory analysis, we examined the potential of the extracted histologic features for predicting cancer status (benign vs. invasive biopsy diagnosis) among women with high and low fibroglandular volume. Firstly, the patient population was stratified by fibroglandular volume (high vs. low), using the median cut point of global (34.4%) and localized (40%) fibroglandular volume from the training population. For this analysis, all in-situ diagnoses were excluded from both model training and testing. Thus the cancerous group was restricted to biopsy diagnoses of invasive carcinoma and the benign group included diagnoses of non-proliferative and proliferative benign breast disease (with and without atypia). Using the 37 features previously extracted from the convolutional neural network output, a random forest classifier was trained to predict cancer status separately among women with high and low fibroglandular volume. The classifier performance for cancer status prediction was assessed using area under the receiver-operating characteristic (ROC) curve (AUC) analysis on the probabilities generated by the random forest classifier. 95% confidence intervals (CIs) were generated using a patient-stratified percentile bootstrapping method.^39^ ROC curves of the cancer detection systems among patients with high and low global or localized % fibroglandular volume were compared using the bootstrap method in R package “pROC”, which computes, stores and compares the AUC of each ROC curve.^40^

## Supplementary information

Supplemental Material

Supplemental Material

## Data Availability

The datasets supporting Fig. 2, Tables 2 and 3 and Supplementary Table 2 of the published article are publicly available in the figshare repository, 10.6084/m9.figshare.9786152.^41^ The raw datasets generated and analysed during this study, and datasets supporting Fig. 3, Table 1 and Supplementary Table 1 of the published article are not publicly available to protect patient privacy, but de-identified data can be made available on request from Dr. Gretchen L. Gierach, as described in the figshare data record above.

**Fig. 2:**
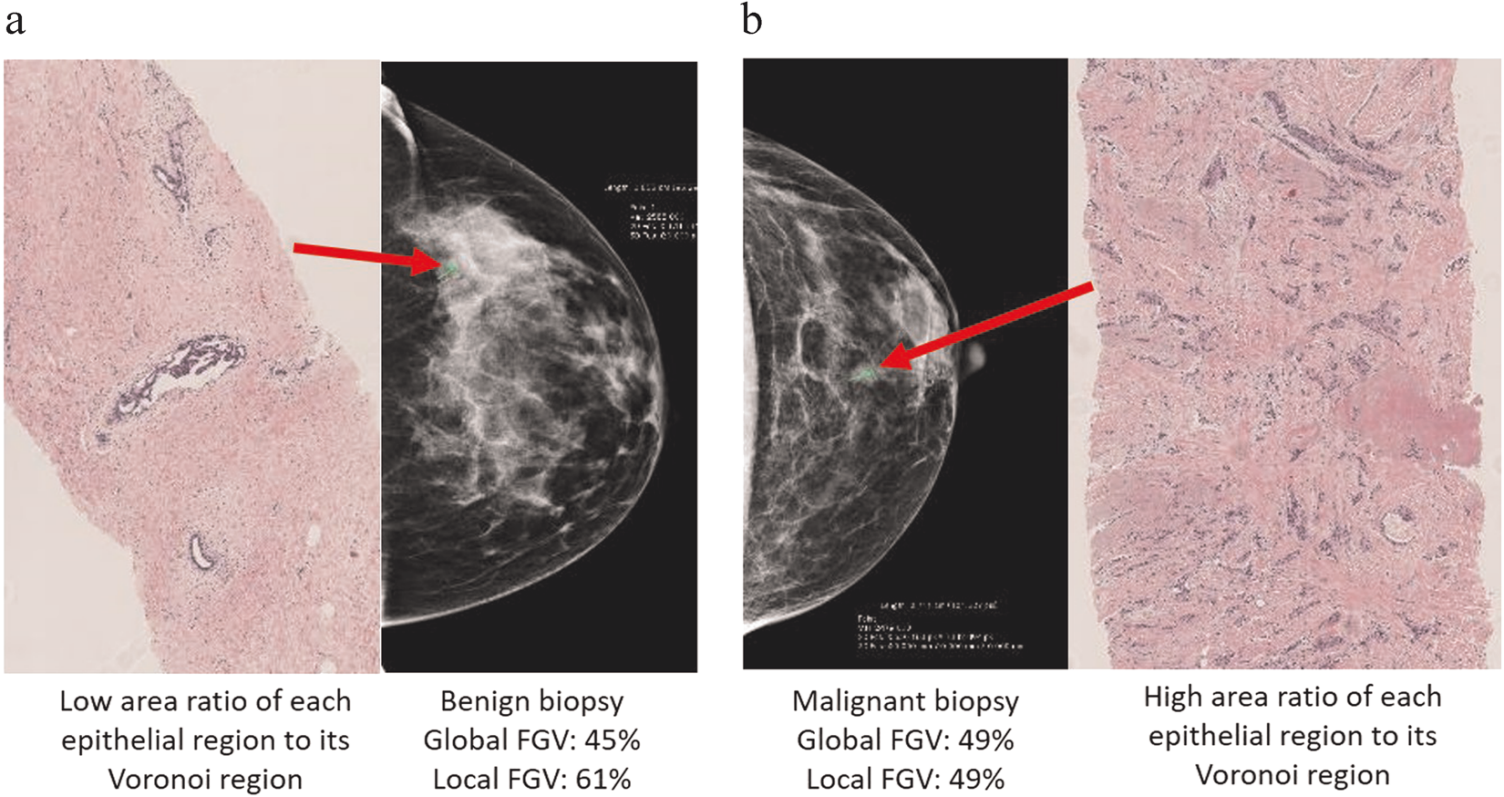
**a**, **b** Representative histological whole slide H&E images of breast biopsies and corresponding full-field digital mammograms from patients with similar radiological global fibroglandular volume but whose biopsies yielded different diagnoses of atypical ductal hyperplasia **a** and invasive carcinoma **b**

**Fig. 3:**
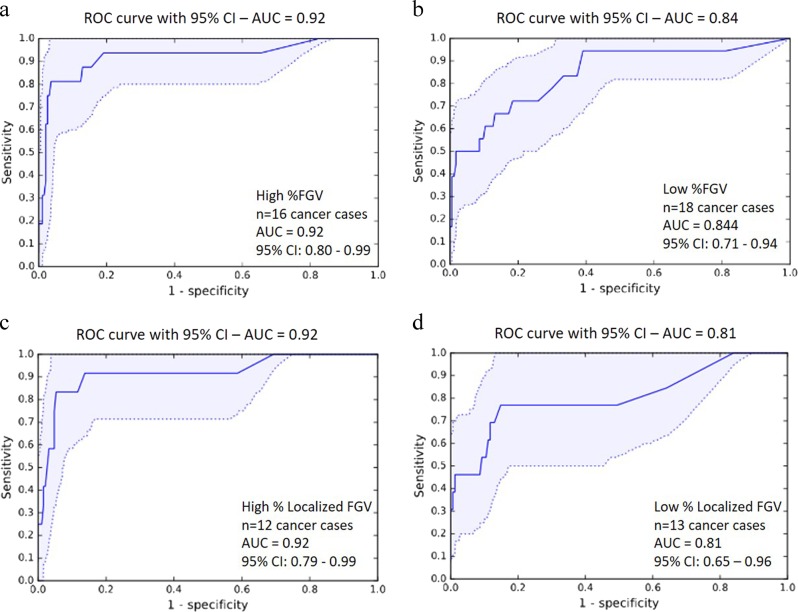
ROC curves (AUC with 95% confidence intervals) for the prediction of invasive cancer among women with high **a** and low **b** percent global fibroglandular volume, high **c** and low **d** percent localized fibroglandular volume. *AUC* area under the curve, *ROC* receiver-operating characteristic **a**, **b** Representative histological whole slide H&E images of breast biopsies and corresponding full-field digital mammograms from patients with similar radiological global fibroglandular volume but whose biopsies yielded different diagnoses of atypical ductal hyperplasia **a** and invasive carcinoma **b** ROC curves (AUC with 95% confidence intervals) for the prediction of invasive cancer among women with high **a** and low **b** percent global fibroglandular volume, high **c** and low **d** percent localized fibroglandular volume. *AUC* area under the curve, *ROC* receiver-operating characteristic

## Code availability

The code developed during the current study are available upon reasonable request.
