# Supplementary material for: Application of convolutional neural networks to breast biopsies to delineate tissue correlates of mammographic breast density
Source: NPJ Breast Cancer. 2019 Nov 19;5:43. doi: 10.1038/s41523-019-0134-6 (PMC6864056; doi:10.1038/s41523-019-0134-6)
Supplement: Supplementary file 2 — Supplemental Material [file 41523_2019_134_MOESM2_ESM.pdf]

Supplementary Figure 1 – Representative biopsy slide showing the CNN model classification of breast biopsy tissue and the output whole slide image maps of epithelium (red), stroma (orange) and fat (green) tissues. Performance of the classification has been previously described. CNN, convolutional neural network.

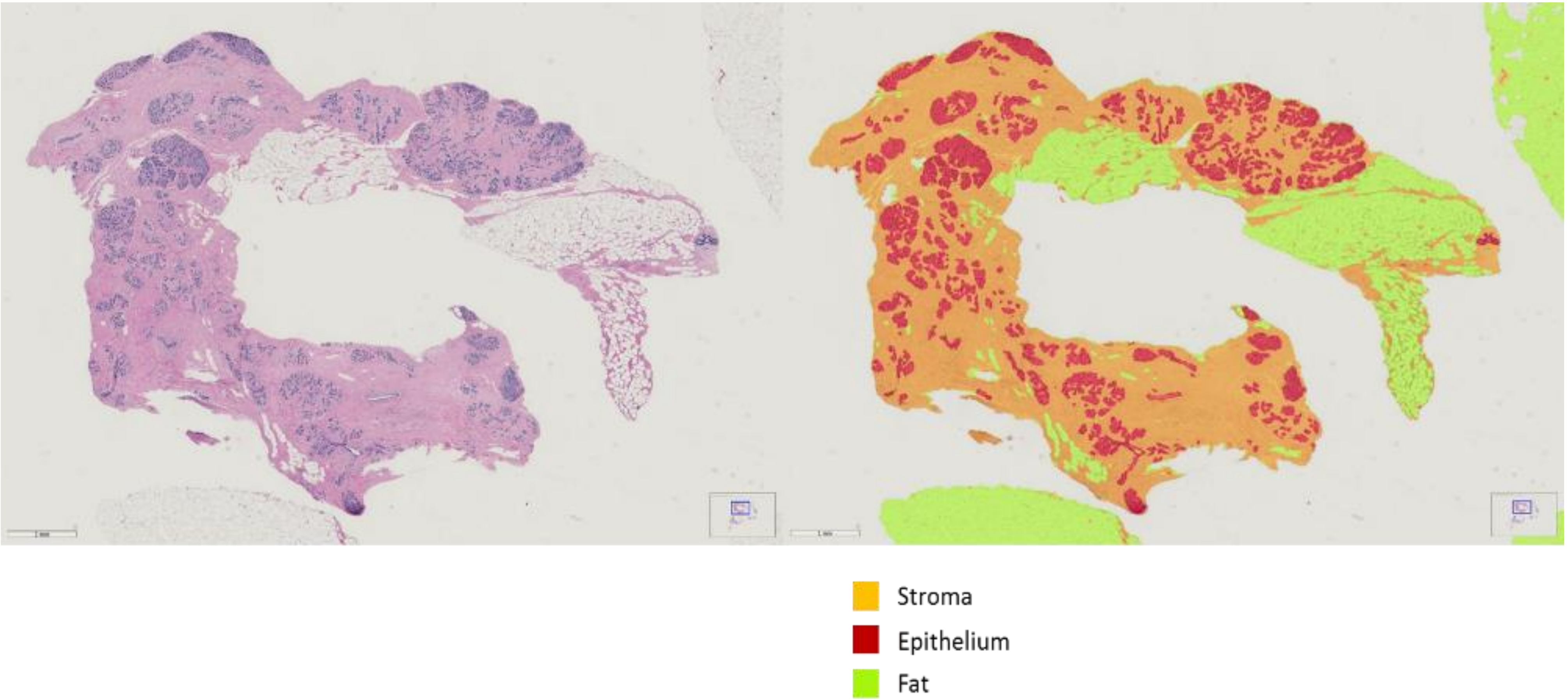

Supplementary Figure 2 – Representative Gini index output for the random forest prediction of features associated with global (A) and localized (B) % mammographic density. The top 10 features from each model prediction performed are listed in Table 2. MD, mammographic density.

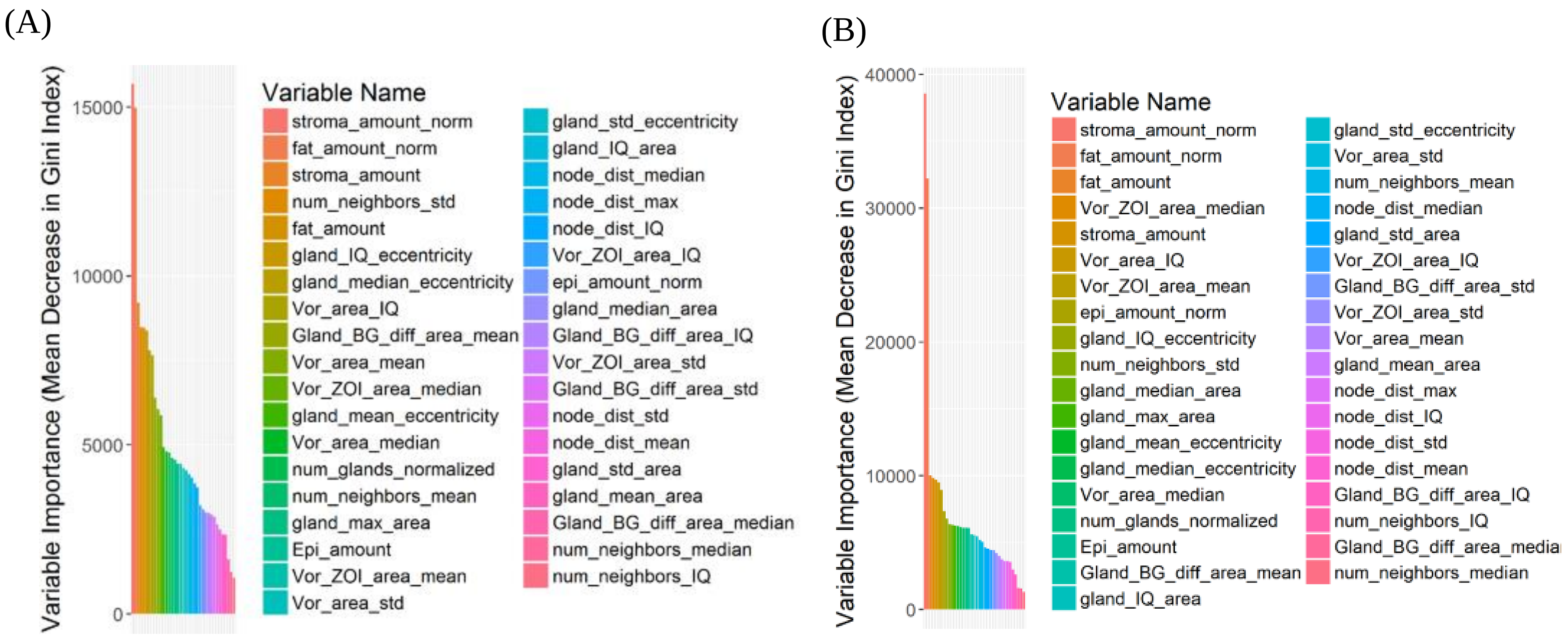

Supplemental Table 1 – Distribution (median (range)) and description of the 37 histologic features extracted from the CNN deep learning output in the H&E stained whole slide images from the training and testing sets

Feature Number	Feature Name	Feature Description	Training WSI (n=1,587)^			Testing WSI (n=454)*		
			Median	Minimum	Maximum	Median	Minimum	Maximum
Global tissue								
1	Fat amount (μm^2)	Total fat amount on slide	114842.25	361.71	937939.01	129548.31	1018.08	1095802.50
4	Fat amount normalized (%)	Total fat amount on slide normalized to total tissue area	46.00	1.00	99.50	48.93	2.27	99.90
2	Stroma amount (μm^2)	Total stroma amount on slide	111813.25	320.83	890662.26	119985.79	93.27	737085.75
5	Stroma amount normalized (%)	Total stroma amount normalized on slide normalized to total tissue area	47.00	0.00	95.50	47.68	0.10	96.74
3	Epithelium amount (μm^2)	Total epithelium amount on slide	8330.22	0.00	258420.18	7554.49	0.00	175299.91
6	Epithelium amount normalized (%)	Total epithelium amount normalized on slide normalized to total tissue area	3.00	0.00	87.00	2.42	0.00	52.86
Morphology								
7	Epithelial regions (mean μm^2)	Area of epithelial regions (mean)	164.91	0.00	3737.84	161.03	0.00	3136.35
8	Epithelial regions (median μm^2)	Area of epithelial regions (median)	98.89	0.00	5243.08	96.51	0.00	497.29
9	Epithelial regions (SD μm^2)	Area of epithelial regions (standard deviation)	162.05	0.00	10956.02	163.27	0.00	5955.21
10	Epithelial regions (IQ μm^2)	Area of epithelial regions (inter-quartile range)	113.15	0.00	6873.88	112.85	0.00	4858.93
11	Epithelial regions (max)	Area of epithelial regions (max)	822.32	0.00	63895.16	794.65	0.00	36972.93
12	Ecc epi regions (mean)	Eccentricity of epithelial regions (mean)	0.82	0.00	0.99	0.82	0.00	0.99
13	Ecc epi regions (median)	Eccentricity of epithelial regions (median)	0.85	0.00	0.99	0.85	0.00	0.99
14	Ecc epi regions (SD)	Eccentricity of epithelial regions (standard deviation)	0.13	0.00	0.29	0.13	0.00	0.26
15	Ecc epi regions (IQ)	Eccentricity of epithelial regions (inter-quartile range)	0.16	0.00	0.38	0.16	0.00	0.27
Spatial arrangement of the epithelial regions (Area-Voronoi diagram)								
16	Voronoi area (mean μm^2)	Area of Voronoi cells (mean)	2067.18	0.00	1737265.18	3170.49	0.00	855885.69
17	Voronoi area (median μm^2)	Area of Voronoi cells (median)	764.23	0.00	1737265.18	933.16	0.00	88318.27
18	Voronoi area (SD μm^2)	Area of Voronoi cells (standard deviation)	2650.52	0.00	1637996.03	2836.76	0.00	1707579.86
19	Voronoi area (IQ μm^2)	Area of Voronoi cells (inter-quartile range)	869.38	0.00	1737360.91	1145.37	0.00	334200.62
20	Ratio epi to Voronoi (mean)	Area ratio of each epithelial region to its Voronoi region (mean)	0.18	0.00	1.00	0.16	0.00	0.54
21	Ratio epi to Voronoi (median)	Area ratio of each epithelial region to its Voronoi region (median)	0.16	0.00	1.00	0.13	0.00	0.61
22	Ratio epi to Voronoi (SD)	Area ratio of each epithelial region to its Voronoi region (standard deviation)	0.11	0.00	0.50	0.11	0.00	0.29
23	Ratio epi to Voronoi (IQ)	Area ratio of each epithelial region to its Voronoi region (inter-quartile range)	0.12	0.00	1.00	0.12	0.00	0.53
24	Ratio epi to non-epi (mean)	Ratio of epithelial region area and non-epithelial gland area (mean)	0.98	0.00	1.00	0.98	0.00	1.00
25	Ratio epi to non-epi (median)	Ratio of epithelial region area and non-epithelial gland area (median)	1.00	0.00	1.00	1.00	0.00	1.00
26	Ratio epi to non-epi (SD)	Ratio of epithelial region area and non-epithelial gland area (standard deviation)	0.03	0.00	0.49	0.03	0.00	0.19
27	Ratio epi to non-epi (IQ)	Ratio of epithelial region area and non-epithelial gland area (inter-quartile range)	0.01	0.00	1.00	0.01	0.00	0.22
Spatial arrangement of the epithelial regions (Delaunay Triangulation)								
28	Neighbors (mean number)	Number of epithelial neighbors (mean)	4.25	0.00	5.78	4.18	0.00	5.64
29	Neighbors (median number)	Number of epithelial neighbors (median)	4.00	0.00	6.00	4.00	0.00	6.00
30	Neighbors (SD number)	Number of epithelial neighbors (standard deviation)	1.22	0.00	1.87	1.20	0.00	1.74
31	Neighbors (IQ number)	Number of epithelial neighbors (inter-quartile range)	1.50	0.00	3.00	1.50	0.00	3.00
32	Neighbor distances (mean μm)	Distances between epithelial regions (mean)	1058.52	0.00	2959.99	1146.19	0.00	3137.12
33	Neighbor distances (median μm)	Distances between epithelial regions (median)	918.22	0.00	2925.20	1019.37	0.00	3497.44
34	Neighbor distances (SD μm)	Distances between epithelial regions (standard deviation)	536.99	0.00	1458.29	587.24	0.00	1816.69
35	Neighbor distances (IQ μm)	Distances between epithelial regions (inter-quartile range)	650.84	0.00	2751.58	702.43	0.00	2582.10
36	Neighbor distances (max μm)	Distances between epithelial regions (max)	2761.78	0.00	4984.75	2972.44	0.00	4988.39
37	Glands per cluster (average number)	Average number of glands in each biopsy cluster	12.75	0.00	303.00	13.00	0.00	126.33

^training set included target and non-target slides
*among non-target slides only
CNN: convolutional neural network; ecc: eccentricity; epi: epithelial; H&E: hematoxylin and eosin; IQ: interquartile; SD: standard deviation; WSI: whole slide image

Supplementary Table 2 – Summary of top 10 ranked histologic features identified in the random forest model that included BMI for the prediction of global volumetric and localized % fibroglandular volume

Feature Name	All Samples (including BMI) Rank of feature importance (including BMI)		All premenopausal samples (including BMI) Rank of feature importance (Premenopausal)		All postmenopausal samples (including BMI) Rank of feature importance (Postmenopausal)	
	Predicted model: global FGV (%)	Predicted model: localized FGV (%)	Predicted model: global FGV (%)	Predicted model: localized FGV (%)	Predicted model: global FGV (%)	Predicted model: localized FGV (%)
BMI	1	1	1	1	1	1
Global tissue amount						
Fat amount (µm^2)	5	6	-	5	4	7
Fat amount normalized (%)	2	2	3	2	6	4
Stroma amount (µm^2)	4	5	5	3	3	3
Stroma amount normalized (%)	3	3	2	4	2	2
Epithelium amount (µm^2)	-	-	-	-	-	-
Epithelium amount normalized (%)	-	7	-	-	-	9
Morphology						
Epithelial regions (mean µm^2)	-	-	-	-	-	-
Epithelial regions (median µm^2)	-	-	8	-	-	10
Epithelial regions (max µm^2)	-	-	10	-	-	-
Epithelial regions (SD µm^2)	-	-	-	-	-	-
Epithelial regions (IQ µm^2)	-	-	-	10	-	-
Ecc epi regions (mean)	-	8	6	-	-	-
Ecc epi regions (median)	8	-	-	7	7	-
Ecc epi regions (SD)	-	-	-	-	9	-
Ecc epi regions (IQ)	-	9	-	-	-	-
Spatial arrangement of the epithelial regions (Area-Voronoi diagram)						
Voronoi area (mean µm^2)	6	-	-	-	-	-
Voronoi area (median µm^2)	7	-	-	6	5	8
Voronoi area (SD µm^2)	-	-	-	9	-	-
Voronoi area (IQ µm^2)	10	-	9	-	8	-
Ratio epi to Voronoi (mean)	-	-	-	-	-	6
Ratio epi to Voronoi (median)	-	4	-	-	-	-
Ratio epi to Voronoi (SD)	-	-	7	-	-	-
Ratio epi to Voronoi (IQ)	-	-	-	-	-	-
Ratio epi to non-epi (mean)	9	10	-	8	-	-
Ratio epi to non-epi (median)	-	-	-	-	-	-
Ratio epi to non-epi (SD)	-	-	4	-	-	-
Spatial arrangement of the epithelial regions (Delaunay Triangulation)						
Neighbors (mean number)	-	-	-	-	-	-
Neighbors (median number)	-	-	-	-	-	-
Neighbors (SD number)	-	-	-	-	10	5
Neighbors (IQ number)	-	-	-	-	-	-
Neighbor distances (mean µm)	-	-	-	-	-	-
Neighbor distances (median µm)	-	-	-	-	-	-
Neighbor distances (SD µm)	-	-	-	-	-	-
Neighbor distances (IQ µm)	-	-	-	-	-	-
Neighbor distances (max µm)	-	-	-	-	-	-
Glands per cluster (average numbe	-	-	-	-	-	-

*among benign group only

Note: Only features ranked within the top 10 for prediction of each density measure are included in the table.

Features are ranked numerically and sequentially from 1-10, with 1 representing the most important feature and 10 representing the 10th most important feature.

BMI:body mass index; ecc: eccentricity; epi: epithelial; IQ: interquartile; FGV: fibroglandular volume; SD: standard deviation
